# Chain Versus Ring: Characterization of a *Meta*‐Phenylene Ladder Polymer and Its Octameric Macrocycle

**DOI:** 10.1002/chem.70744

**Published:** 2026-02-09

**Authors:** Paulo D. Nunes Barradas, Hauke J. Jötten, Ngoc B. B. Nguyen, Ullrich Scherf, J. Sérgio de Seixas de Melo

**Affiliations:** ^1^ CQC‐IMS Department of Chemistry University of Coimbra Coimbra Portugal; ^2^ Macromolecular Chemistry Group (buwmakro) and Wuppertal Center for Smart Materials and Systems (Cm@S) Bergische Universität Wuppertal Wuppertal Germany

**Keywords:** aromatic ladder polymers, ladder‐type *meta*‐phenylene macrocycle, *meta*‐phenylene ladder polymer, time‐correlated single‐photon counting

## Abstract

A methyl‐substituted ladder *meta*‐phenylene macrocycle (**MeLMMP**) and its corresponding ladder polymer (**MeLPMP**), a *meta*‐analogue of the well‐known ladder‐type poly(*para*‐phenylene), **LPPP**, were synthesized and comprehensively investigated. Both compounds exhibit limited conjugation due to the *meta*‐linked phenylene units, resulting in absorption and emission features shaped by cross‐conjugation. Despite having a longer chain, **MeLPMP** and **MeLMMP** exhibit nearly identical electronic and photophysical behavior, suggesting that the number of repeat units has minimal influence. **MeLPMP** exhibits enhanced vibronic resolution compared to **MeLMMP** due to a broadening of the optical bands of the macrocycle caused by the presence of a mixture of stereoisomers formed during the non‐stereoselective ladderization step. A small amount of fluorenone‐type keto defects produces a weak emission near 500 nm, more evident in the macrocycle, and introduces radiationless decay pathways that compete with fluorescence. This decay pathway, together with the weak π,π* conjugation in these angular compounds, lowers the fluorescence quantum yield when compared with the linear **MeLPPP**, while promoting singlet–triplet conversion and phosphorescence. The data indicate the presence of three chromophoric populations: pristine oligo(*meta*‐phenylene) units; units quenched by nearby keto defects; and intramolecular charge‐transfer complexes (ICTCs) formed between oligo(*meta*‐phenylene) units and keto defects. These findings clarify the photophysics of *meta*‐phenylene ladder systems, showing that the **MeLMMP** macrocycle can act as a structural and electronic model for related ladder polymers.

## Graphical abstract

The synthesis and comprehensive spectral and photophysical analysis of a methyl‐substituted ladder *meta*‐phenylene macrocycle (**MeLMMP**) and its polymer analogue (**MeLPMP**) show that, despite differing topologies (macrocycle vs. polymer), both display nearly identical electronic and photophysical properties due to cross‐conjugation.

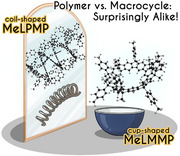

## 1 Introduction

In recent years, the research and development of conjugated macrocyclic systems has increased, mainly due to their potential applications in various types of organic electronic devices and in host–guest chemistry [1, 2, 3, 4]. The electronic and spectroscopic properties of these systems can be modified by varying the size of the macrocycle, incorporating additional structures such as metal–organic frameworks, or introducing heteroatoms and functional groups that facilitate the formation of a donor–acceptor system [4, 5, 6, 7, 8, 9].

Aromatic phenylene units are one of the most used structural motifs for the design of novel conjugated macrocycles, either as single structures or in combination with other functional groups. When used alone, most research on conjugated macrocyclic systems has focused on cyclo‐*para‐*phenylenes, which are the smallest possible armchair carbon nanotubes [10, 11, 12]. Unfortunately, their counterparts, the *meta*‐phenylene macrocycles, have not attracted as much interest. Wu et al. used a synthetic method commonly used for the synthesis of ladder polymers to obtain a *meta*‐phenylene macrocycle and its corresponding ladder structure from dialdehyde monomers [13, 14, 15]. The octamer (an octaphenylene, consisting of four condensed fluorene repeat units) was identified as the preferred ring size. The reduced, saturated octa‐*meta*‐phenylene macrocycle was used as an intermediate to generate the unstable, antiaromatic (oxidized) annulene form and investigate the behavior of the polyradicaloid species [13, 15]. However, to our best knowledge, no detailed spectroscopic or photophysical studies have been reported on these systems.

**FIGURE 1:**
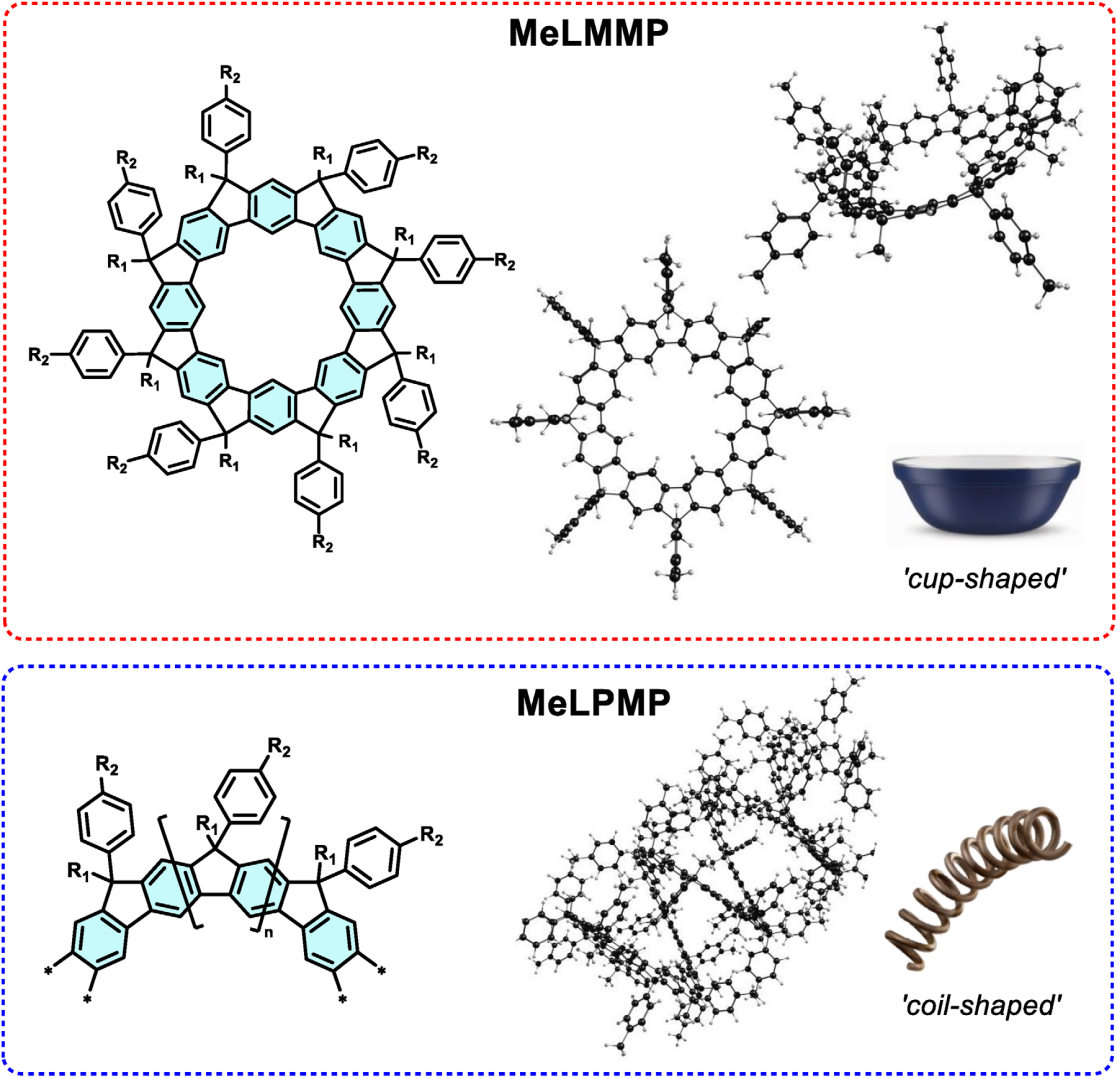
Chemical structures of methyl‐substituted ladder (*meta*‐phenylene) macrocycle (**MeLMMP**) and ladder poly(*meta*‐phenylene) (**MeLPMP**), *R_1_ = methyl, R_2_* = *n*‐decyl. The **MeLMMP** results are accompanied by DFT‐optimized structures of a model octameric macrocycle at the B3LYP/6‐311G (*d, p*) level of theory, and the model polymer **MeLPMP** structure was optimized at the semi‐empirical PM6 level of theory (see Supporting Information for further details).

To study the spectroscopic signature of *meta*‐conjugated macrocycles, a similar ladder *meta*‐phenylene macrocycle was synthesized in this work. However, in this case, a linear phenylalkyl and a methyl (─CH_3_) group were introduced at the methylene bridges to increase the solubility and stability of the compound. The resulting methyl‐substituted ladder(*meta*‐phenylene) macrocycle (**MeLMMP**), which adopts a “cup‐shaped” geometry, was compared with its “coil‐shaped” polymer analogue, the methyl‐substituted ladder poly(*meta*‐phenylene) (**MeLPMP**), as illustrated in Figure 1.

## 2 Results and Discussion

### 2.1 Synthesis and Characterization

**SCHEME 1:**
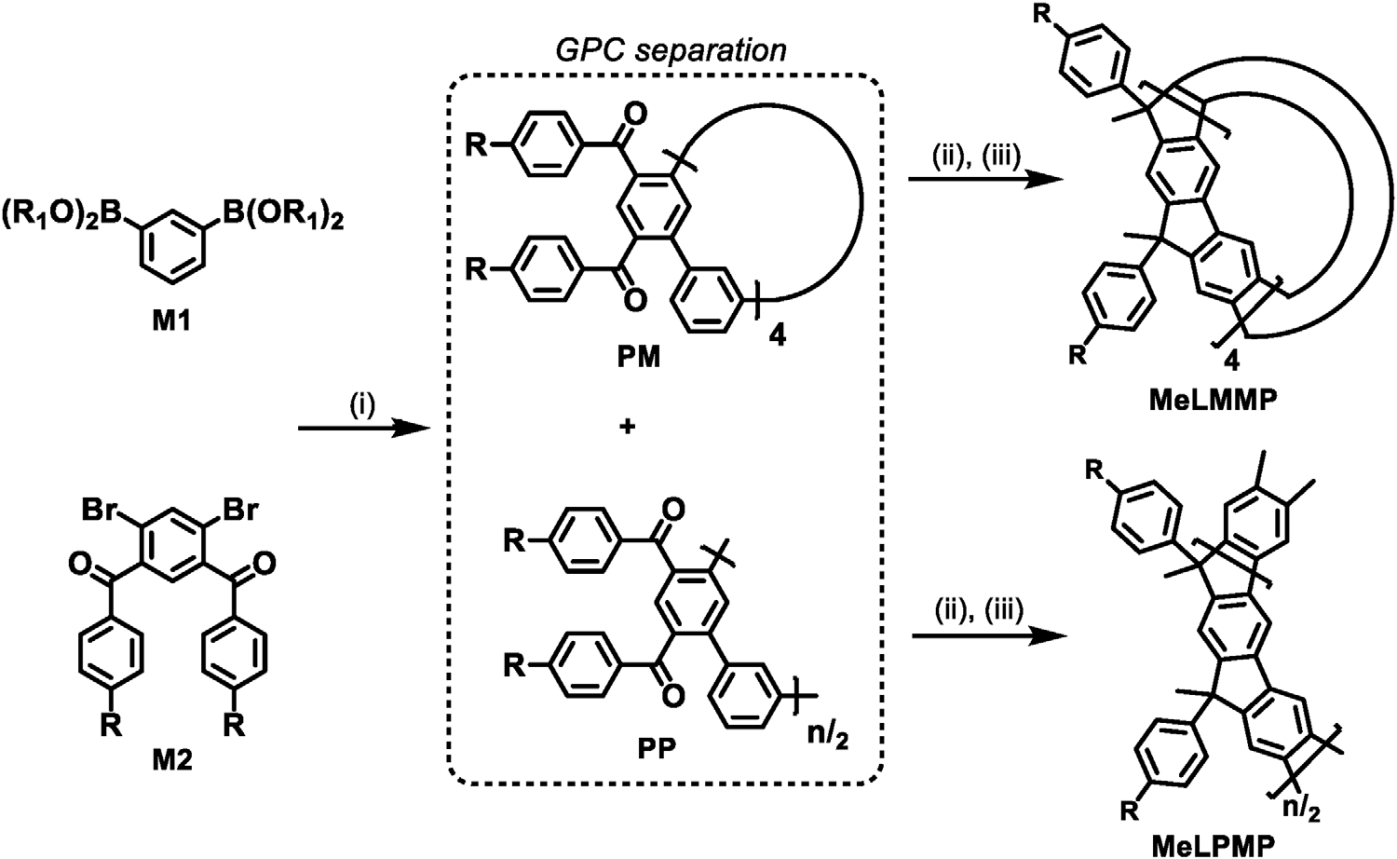
Synthesis of a mixture of precursor polymer PP and precursor macrocycle PM and ladderization of the precursors PP and PM to the final products **MeLPMP** and **MeLMMP**, (R: *n*‐decyl, R_1_‐R_1_: CMe_2_‐CMe_2_); (i) Pd(PPh_3_)_4_, K_2_CO_3_, toluene/H_2_O, 75°C, 72 h, (ii) MeLi, toluene, 0°C, 18 h, and (iii) BF_3_^.^O(C_2_H_5_)_2_, DCM, 18 h.

**MeLPMP** and **MeLMMP** were synthesized in a three‐step procedure starting with a Suzuki‐type cross‐coupling between diboronic ester **M1** and diketone **M2**, forming the mixture of precursors **PP** and **PM** as shown in Scheme 1. The precursors were then separated by recycling GPC (see, Figures S1–S5 and Tables S1 and S2). PP and PM are each methylated with methyllithium (Scheme 1). The intermediate cyclic oligo‐ or polyalcohols are then ladderized, without purification, into the final products, polymer **MeLPMP** (investigated fraction M_n_: 10.2k, DP: ca. 32) and macrocycle **MeLMMP**, for the macrocycle coupled to a second recycling GPC step (Figure S5a, b). Further synthetic details, including NMR spectra, can be found in the Supporting Information (Figures S6 and S7 and Tables S3 and S4).

### 2.2 Steady‐State Photoluminescence

**FIGURE 2:**
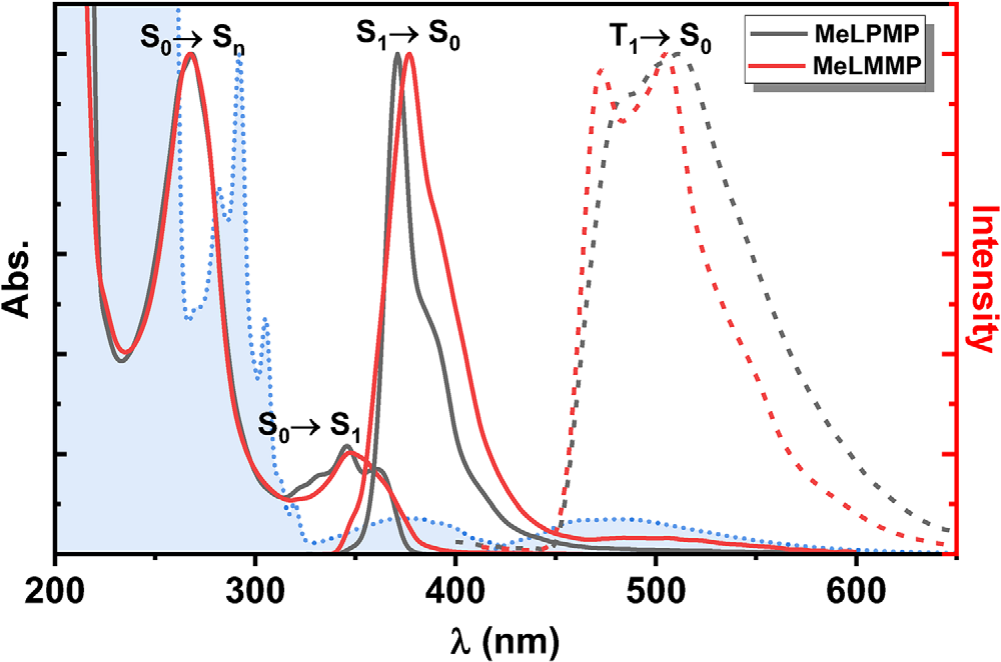
Absorption and fluorescence emission spectra at 293 K, *λ*_exc_ = 310 nm, and (dotted) phosphorescence emission spectra at 77 K, *λ*_exc_ = 350 nm of **MeLPMP** (black lines) and **MeLMMP** (red lines) in MCH. Spectra of 9‐fluorenone (dashed blue) recorded under the same conditions are included as a reference for identifying keto‐defect signatures.

The electronic spectral properties of the macrocycle **MeLMMP** and the polymer **MeLPMP** were investigated in methylcyclohexane solution (Figure 2). Both compounds display similar absorption spectra in solution, characterized by an intense band at 270 nm assigned to transitions from the ground state to higher excited states (*S*_0_→*S*_n_), and a lower‐energy band at ∼350 nm corresponding to the *S*_0_→*S*_1_ transition. **MeLPMP** displays a vibronic structure in the lower‐energy band, absent in the macrocyclic **MeLMMP**. The emission spectra of both systems in solution (Figure 2) were also found to be similar, with a slightly red‐shifted emission maximum observed for **MeLMMP** (*E*_S1_ of 3.28 eV in MCH) relative to **MeLPMP** (*E*_S1_ of 3.34 eV in MCH). For both compounds, the 0–0 transition energy was estimated to be 3.38 eV. Additionally, **MeLMMP** exhibits a distinct low‐intensity emission band at ∼500 nm. This emission matches that of 9‐fluorenone and is attributed to keto defects in the macrocycle, supported by weak C═O stretching bands observed between 1720 and 1800 cm^−1^ in the FTIR spectrum (Figure S8). The low intensity of these IR bands indicates a minor presence of such keto defects [16, 17, 18, 19].

Emission from fluorenone defects has also been reported in related ladder polymers, such as **MeLPPP**, originating from photooxidative degradation reactions during/after the ladderization step and the work‐up procedure [19, 20, 21, 22]. No distinct low‐energy emission band is apparently detected in **MeLPMP**. However, because **MeLPMP** exhibits a higher molar absorptivity coefficient (*ε*), as reported in Table 1, fewer chromophores were present in solutions under the same dilute conditions (Abs ≈ 0.05) employed for comparison between the macrocyclic and polymeric samples. Consequently, the overall concentration of potential keto‐defective units was reduced, leading to a diminished contribution to the emission spectrum. Under these conditions, emission was dominated by defect‐free segments, which are intrinsically more fluorescent, thereby likely masking contributions from the small population of keto‐defective units. Nevertheless, in the samples investigated, **MeLMMP** exhibited a relatively higher ratio of keto‐defective to pristine units.

**TABLE 1:** Relevant spectroscopic (including wavelength absorption, *λ*_abs_, fluorescence emission maxima, *λ*_F_, phosphorescence emission maxima, *λ*_Ph_, Stokes Shift, Δ_SS_, and singlet–triplet energy gap, Δ*E*_S–T_), and photophysical (fluorescence quantum yields, *φ*_F_) parameters for **MeLMMP** and **MeLPMP** in methylcyclohexane (MCH) and toluene at *T* = 298 K.

Compound	Solvent	*λ*_abs_ (nm)	*λ*_F_(nm)	*λ*_Ph_ (nm)	Δ_ss_ (cm^−1^)	*ε* (nM^−1^ L cm^−1^)^a^	Δ*E*_S–T_ (eV)^b^	*φ*_F_
**MeLMMP**	MCH	348/267	377	473, 505	2210	44.5	0.76	0.231 ± 0.007
**MeLPMP**		360/346/268	371	483, 513	824	227.2	0.81	0.207 ± 0.035
**MeLMMP**	Toluene	349	379		2268	45.5		0.275 ± 0.009
**MeLPMP**		359/346	373		1046	230.8		0.256 ± 0.017

^a^ Molar absorptivity coefficient (*ε*) was estimated for **MeLPMP** using a molecular weight (*M*_w_) of 13,000 g/mol, as determined by GPC analysis.

^b^ The Δ*E*_S–T_ value was determined by subtracting the 0–0 transition energy, derived from the absorption and fluorescence spectra (3.38 eV for both compounds), from the onset of the phosphorescence spectra recorded in the same solvent. The resulting values were subsequently converted to electron volts (eV) for standardization.

The triplet states of both **MeLPMP** and **MeLMMP** were also examined. At 77 K, phosphorescence emission was observed for the two compounds (Figure 2). The energy of the lowest triplet state (*T*_1_), obtained from the onset of the phosphorescence spectra, is 2.62 eV for **MeLMMP** and 2.57 eV for **MeLPMP**. This corresponds to an *S*_1_–*T*_1_ singlet–triplet energy gap of ∼0.8 eV for both compounds (Table 1).

**FIGURE 3:**
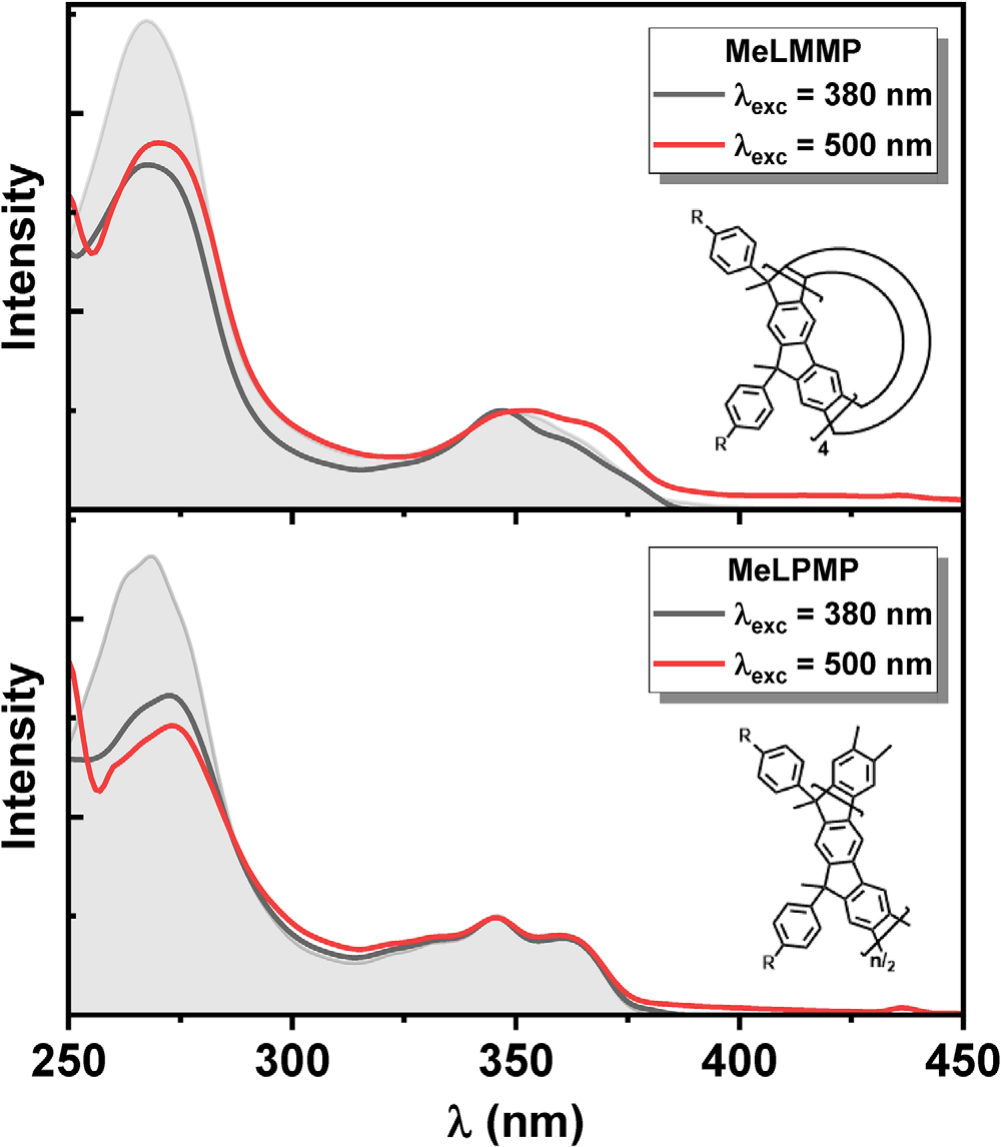
Normalized excitation spectra of (top) **MeLMMP** and (bottom) **MeLPMP** in MCH at 293 K, with emission monitored at 380 nm (black) and 500 nm (red). Normalized absorption spectra (gray‐filled) are included for reference; (*R*: *n*‐decyl).

Existence of keto‐defective sites is further supported by the excitation spectra recorded at 380 nm (nondefective emission) and 500 nm (defective emission, Figure 3). For **MeLPMP**, the spectra are nearly identical at both detection wavelengths, indicating that emission predominantly arises from defect‐free units. In contrast, **MeLMMP** shows distinct excitation spectra profiles when emission (*λ*_em_) is collected at 380 and 500 nm, confirming that emission at longer wavelengths is directly associated with fluorenone‐like defects. These findings highlight the greater sensitivity of the macrocycle to defect‐state emission compared to its polymeric analogue.

As shown in Table 1, the fluorescence quantum yields (*ϕ_F_*) of **MeLMMP** and **MeLPMP** are similar, yet roughly four times lower than those reported for linear ladder‐type poly(*para*‐phenylene), **MeLPPP** [23]. This decrease is attributed to the angular and helical geometries of the macrocyclic and polymeric frameworks, which restrict π‐conjugation relative to the more efficient planar configuration. Additionally, the presence of keto defects introduces non‐bonding orbitals that enable *n*→π* transitions, providing alternative radiationless decay pathways that compete with fluorescence.

Although spectroscopic data indicate a higher concentration of keto defects in the macrocycle **MeLMMP**, it still displays a slightly higher *ϕ*_F_ in solution compared to the polymer **MeLPMP** (Table 1). This behavior suggests that in **MeLPMP**, intramolecular interactions, particularly the close spatial arrangement of chromophoric units along the helical backbone (see Figure 1), may promote additional radiationless deactivation pathways, thereby reducing the fluorescence efficiency. The smaller Stokes shift (Δ_ss_) observed in **MeLPMP**, when compared with the macrocycle **MeLMMP**, further supports this interpretation and is consistent with the enhanced structural rigidity associated with the helical backbone of the polymer. A dependency of the Δ_ss_ on the solvent was also observed, with the largest Δ_ss_ recorded in toluene. The increase in the Stokes shift from cyclohexane to toluene is attributed to the enhanced stabilization of the excited state due to increased solvent polarity and specific pi‐solvent interactions; these factors lower the emission energy while minimally affecting the absorption transition.

### 2.3 Statistical Analysis of Keto Defects in the Macrocycle **MeLMMP**

Ladder‐type conjugated structures are typically associated with high structural rigidity, which often results in well‐resolved absorption and emission spectra. The polymer **MeLPMP** exhibits this expected behavior. Despite its polymeric nature, its rigid helical backbone gives rise to vibronically structured absorption and sharp fluorescence emission. In contrast, the macrocyclic compound **MeLMMP** displays significantly broadened absorption and emission spectra. Although this may seem counterintuitive given its smaller size and well‐defined molecular architecture, the broadening is consistent with inhomogeneous spectral features arising from a distribution of structural isomers within the sample. Indeed, these isomeric species subtly modify the local electronic environments, contributing to the experimentally observed spectral broadening. In particular, the intramolecular nucleophilic attack on the carbonyl group that forms the bridgehead fluorene structure can occur in two different orientations, yielding two possible stereoisomeric positions for the methyl group at each bridgehead. This absence of stereochemical control results in multiple isomers, particularly in small macrocycles such as **MeLMMP**, where steric constraints are less significant.

**FIGURE 4:**
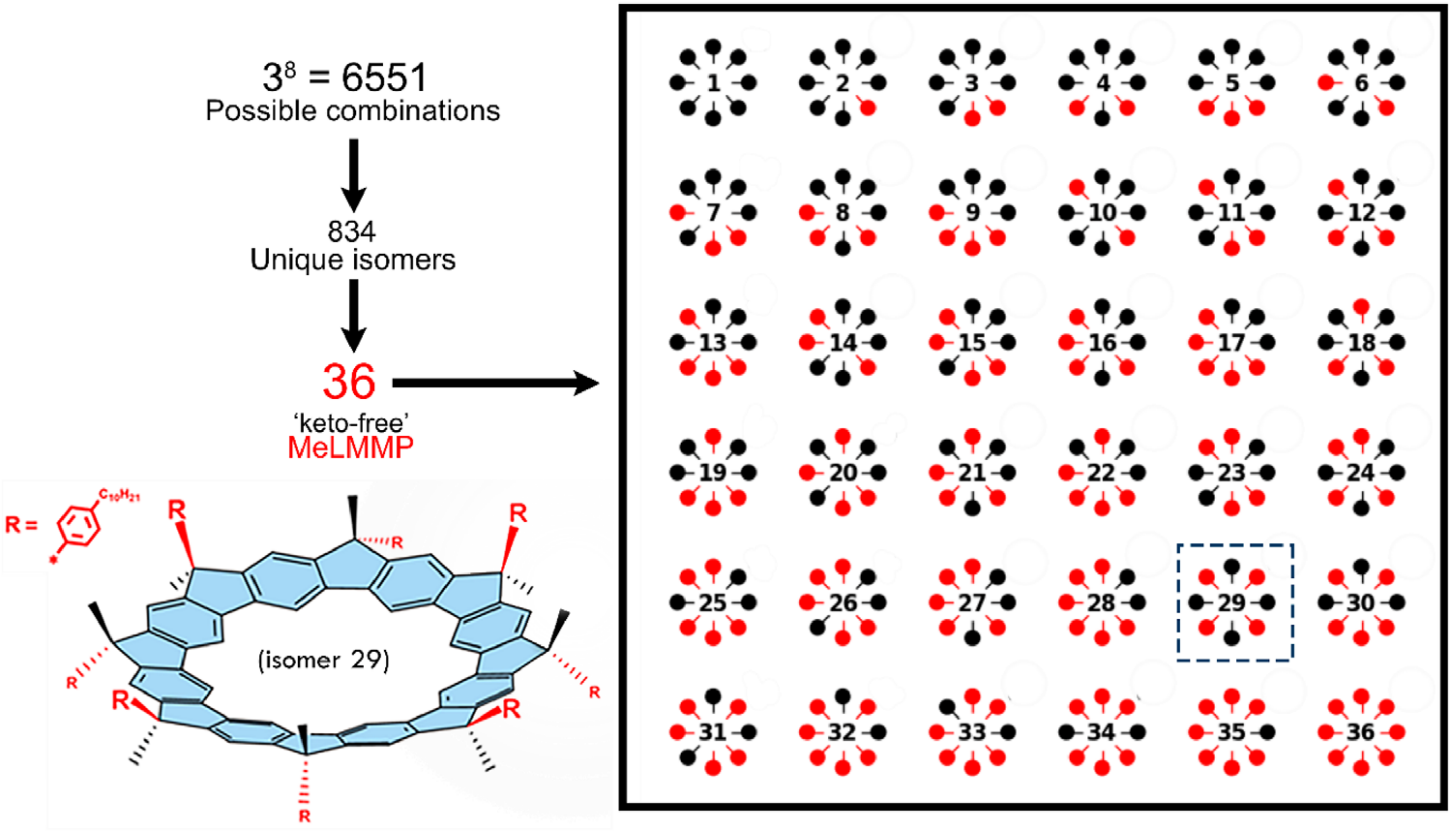
Schematic representation of all possible isomers of **MeLMMP**, highlighting the 36 keto‐defect‐free isomers. The Figure also shows a visual model of isomer 29, identified as the most stable structure based on semi‐empirical and TDDFT calculations (see Supporting Information for further details).

In **MeLMMP** with its eight bridgehead positions, this results in 2^8^ = 256 possible stereoisomers for a macrocycle with no keto defects: the nondefective macrocycle. The inclusion of keto defects further increases this complexity. Indeed, when accounting for the presence of possible defect sites, the theoretical number of structural permutations increases to 3^8^. However, the cup‐shaped geometry of **MeLMMP** (Figure 1) imposes a high degree of molecular symmetry. In the absence of substituents, the macrocycle can be approximated by a *C_8v_* point group, which significantly reduces the number of unique stereoisomers due to indistinguishability under symmetry operations. As detailed in the Supporting Information, symmetry analysis indicates that only 834 distinct isomers are possible when keto defects are taken into account. Among these, 36 correspond to fully nondefective stereoisomers (Figure 4). Furthermore, assuming that the probability of forming a defective unit is equal to that of a defect‐free unit, the most probable stereoisomeric configurations are predicted to contain two to three defects per macrocycle (see Figure S10 and related discussion). Therefore, because keto‐defect formation is expected to occur to a lower extent under realistic conditions, the average number of defects per macrocycle is anticipated to be below the probabilistic expectation, that is, fewer than two.

Keto defects in methyl‐bridged phenylenes generally arise after the post‐polymerization and work‐up steps, resulting from photooxidative degradation, rather than from direct oxidation via (hydro)peroxide formation as observed in H‐bridged polyfluorenes [19, 20, 21, 22]. Keto defects are generally less frequently observed for methyl‐substituted bridgehead carbons. In addition, previous studies on open‐angle ladder‐type *meta*‐phenylenes [24], both with and without keto defects and with varying lengths of phenyl conjugation, which can be considered model compounds for **MeLMMP** based on their structural and spectroscopic similarities, have provided further insight. These studies showed that nondefective *meta*‐phenylenes exhibit a progressive bathochromic shift in fluorescence emission maxima with increasing numbers of phenyl rings. In particular, the largest nondefective compound examined, containing seven phenyl rings, displayed an emission maximum near 371–375 nm, which is consistent with the emission of **MeLMMP**, comprising eight phenyl rings and exhibiting emission in the 377 to 379 nm region depending on the solvent. In contrast, keto‐defective model compounds showed emission centered around 500 nm, in agreement with the low‐energy band observed in the **MeLMMP** emission spectrum. Therefore, despite the presence of some keto‐defective units, the majority of stereoisomers of **MeLMMP** is inferred to be keto‐free, while the fraction containing keto defects is limited and, based on comparison with model compounds and the low extent of oxidation typically observed in methyl‐bridged polyfluorenes, is expected to correspond to at most one defect per macrocycle.

### 2.4 Time‐Resolved Measurements

Fluorescence decay times of the macrocycle **MeLMMP** and the polymer **MeLPMP** were measured in different solvents at emission wavelengths (*λ*_em_) of 380 and 500 nm. The resulting decays were subjected to global analysis. As summarized in Table 2, both systems in solution exhibited tri‐exponential decay behavior.

**TABLE 2:** Fluorescence emission lifetimes (*τ*_F_) and phosphorescence decay lifetimes (*τ*_phosp_) of **MeLMMP** and **MeLPMP** in different solvents under atmospheric conditions. The percentual fractional intensity for each decay time (%*f*_i_) and amplitude average lifetime (<*τ*_F_>) are also presented.

Compound	Solvent	*λ*_em_ (nm)	*τ*_F_ *,_i_* (%*f_i_*) (ns)						<*τ*_F_> (ns)	*τ*_phosp_,*_i_* (%*f_i_*) (s)	
**MeLMMP**	MCH	380 500	1.2	(12) (33)	3.4	(86) (45)	10.5	(2) (22)	2.8 2.4	0.29 (10)	3.7 (90)
	Toluene	380 500	1.6	(23) (64)	3.4	(75) (17)	10	(2) (18)	2.7 2.1		
**MeLPMP**	MCH	380 500	1.0	(30) (1)	2.6	(69) (43)	10.8	(1) (55)	1.7 4.3	0.12 (18)	0.68 (82)
	Toluene	380 500	1.1	(39) (8)	2.5	(60) (55)	10.7	(1) (37)	1.7 4.3		

*Note*: Fluorescence lifetimes in solution were obtained by global analysis of the fluorescence decays by the sum of discrete exponentials $I\left(t\right)=\sum_{i=1}^{n}a_{i}e^{\left(-t/\tau_{i}\right)}$ with *λ*_exc_ = 339 nm. Phosphorescence lifetime was obtained at 77 K, monitored at 500 nm with *λ*_exc_ = 350 nm. Fractional intensity in percentage $\left(\%f_{i}\right)=a_{i}\tau_{i}/\sum_{i}^{j}a_{i}\tau_{i}\times 100$. Amplitude average lifetime $\left\langle \tau_{F}\right\rangle =\sum_{i}^{j}a_{i}\tau_{i}/\sum a_{i}$ [25].

In solution, the fluorescence decay times of **MeLMMP** and **MeLPMP** are generally comparable, with the polymeric **MeLPMP** consistently exhibiting slightly shorter decay times in all solvents. This decrease in decay time values in the polymeric **MeLPMP** can be attributed to self‐quenching caused by the spatial proximity of other chromophoric units, because of its helical macromolecular structure (see Figure 1). Therefore, these results indicate that the macrocycle **MeLMMP** can serve as a model chromophoric unit of the polymeric **MeLPMP**. Accordingly, the length of the primarily conjugated segments in **MeLPMP** is well matched by the eight bridged *meta*‐phenylene units as present in **MeLMMP**, within the macrocycle arranged into an angular, cup‐shaped ladder‐type configuration (Figure 1).

Further analysis of the fluorescence decays shows that at 380 nm, the intermediate decay time component, ranging from 2.5 to 3.4 ns, dominates and is attributed to the fluorescence of undisturbed ladder‐type oligo(*meta*‐phenylene) units. The longer values of these decay time components, by comparison with the analogous linear *para*‐phenylene ladder polymer **MeLPPP** (*τ*_F_ ≈ 0.34 ns) [23], can be explained by the different electronic and conformational structure of the studied *meta*‐phenylene ladder compounds, whose cross‐conjugated units form cup‐shaped or helical geometries, which hinder a wider energy delocalization due to the lack of extended planarization.

At 500 nm, corresponding to the spectral region influenced by keto defects, the relative contribution of the longer decay time components generally increases both for polymer **MeLPMP** and macrocycle **MeLMMP**, and for the macrocycle **MeLMMP**, the contribution of the shorter decay time component also increases.

This suggests that additional decay pathways are associated with the presence of keto defects. Previous studies on non‐ladder‐type conjugated polyfluorene/fluorenone (PL/FL) copolymers have attributed the longest decay component observed in these systems (∼6 ns) to the formation of intramolecular charge‐transfer complexes (ICTCs) between defective and nondefective units [26]. Similarly, the long emission lifetime component observed for the keto defect in **MeLMMP** and **MeLPMP** (∼10 ns) can be attributed to the presence of an ICTC formed between fluorenone defects and nearby oligo(*meta*‐phenylene) units. The shorter decay time component is, in turn, attributed to the fluorescence of oligo(*meta*‐phenylene) units quenched by their proximity to keto defects [26].

**FIGURE 5:**
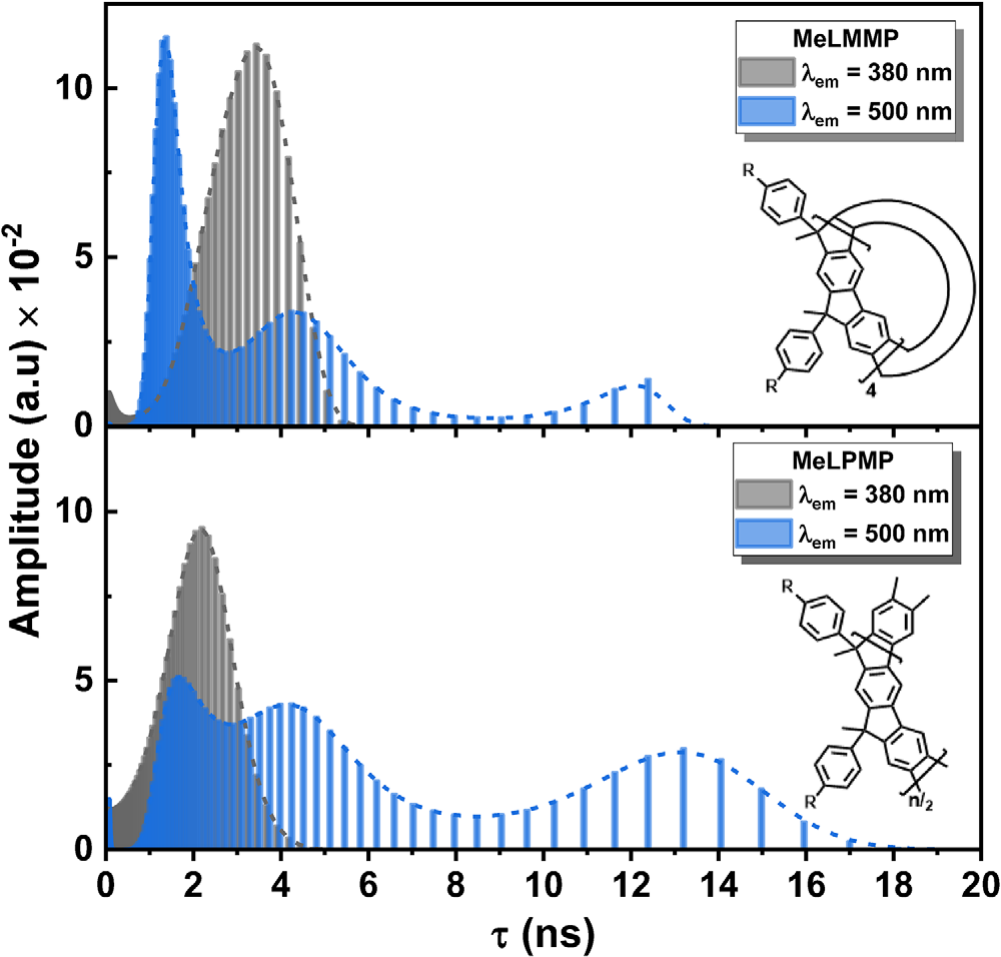
Lifetime decay distribution obtained by MEM analysis of (top) **MeLMMP** and (bottom) **MeLPMP** in MCH, black and blue distributions are referred to the individual analysis with *λ*_em_ = 380, and 500 nm, respectively; (*R*: *n*‐decyl).

In polymeric or macromolecular systems, fluorescence decays often deviate from exponential decay behavior because inter‐ and intramolecular interactions can affect the local chemical environment of the emissive species. Furthermore, these interactions can often result in the inclusion of an additional exponential term that is not necessarily associated with a specific emitting species. The analysis with the maximum entropy method (MEM) can then be used to study the distribution of fluorescence lifetimes. Independent lifetime distribution analysis using the MEM, shown in Figure 5, confirmed the presence of three distinct emissive species, consistent with the results obtained from the previous analysis with the sum of discrete exponentials.

MEM analysis at 500 nm reveals contributions from three distinct species: a long‐lived component (10–16 ns) and two shorter components in the 1–3 ns (also present at 380 nm) and 4–6 ns range. This supports the interpretation that the fluorescence decays originate from different emissive species. The shortest decay component (∼1–3 ns) is attributed to oligo(*meta*‐phenylene) chromophoric units that undergo quenching due to their immediate proximity to adjacent keto defects. This contribution is particularly prominent in the MEM analysis of **MeLMMP**. Given that a higher effective population of keto‐defective units is present in the macrocyclic sample relative to **MeLPMP**, a greater fraction of the backbone chromophores is subjected to this quenching mechanism. Consequently, this results in a significantly higher relative intensity of the short‐lived decay component in the macrocycle compared to its longer‐lived counterparts and the polymeric **MeLPMP**. The intermediate component (∼4–6 ns) is attributed to pristine oligo(*meta*‐phenylene) units unaffected by defects. Finally, the long‐lived component (10–15 ns) is associated with fluorenone defects, incorporated into ICTCs with neighboring *meta*‐phenylene segments.

DFT and TDDFT calculations were performed on a model structure of **MeLMMP,** without keto‐defects. These calculations revealed that the lowest singlet excited state (*S*_1_) is predominantly characterized by a HOMO→LUMO transition, as shown in Figure S13. This corresponds to a π→π* transition. For polymeric or macrocyclic segments in direct proximity to keto‐defect sites, a similar π→π* character of the *S*_1_ state is expected. The predominant presence of pristine units stabilizes this state, mirroring the behavior observed for isolated 9‐fluorenone in polar environments [27]. This stabilization explains why the longest fluorescence decay lifetime is associated with the keto‐defective units in the TCSPC measurements.

Time‐resolved phosphorescence measurements (at 77 K) revealed bi‐exponential decays for both compounds (Table 2), with the shorter lifetime accounting for less than 20% of the total emission. Under identical experimental conditions, no detectable phosphorescence was observed for the reference compound 9‐fluorenone. For both materials, the shorter phosphorescence lifetimes fall within the millisecond regime, with values of approximately 290 and 120 ms for **MeLMMP** and **MeLPMP**, respectively. In contrast, the second and dominant phosphorescence component observed for **MeLMMP** exhibits a substantially longer lifetime of 3.7 s. This shows that the *T*_1_ excited state is predominantly of π, π* character, making the *T*_1_→*S*_0_ (^3^π, π*→^1^π, π) transition both spin‐ and symmetry‐forbidden. Given that the *S*_1_ state is also of π, π* character, the efficiency of the *S*_1_⇝*T*_1_ intersystem crossing process is low, consistent with El‐Sayed's rules [28, 29]. In the case of **MeLPMP**, the longest phosphorescence decay time is approximately 680 ms, which is notably shorter than that observed for the **MeLMMP** macrocycle. This behavior is attributed to more efficient triplet energy migration along the polymeric backbone. Even at low temperatures, this process accelerates nonradiative decay by increasing the exciton diffusion length, thereby enhancing the probability of encounters with defects that act as quenching traps. This interpretation is supported by the larger contribution of the shortest lifetime component to the phosphorescence decay of **MeLPMP** compared with **MeLMMP**, despite the latter containing a higher fraction of keto defects. Nevertheless, the observation of long‐lived species indicates that phosphorescence in both **MeLMMP** and **MeLPMP** originates from pristine ladder‐type oligo(*meta*‐phenylene) repeat units and from segments located in proximity to keto defects, rather than from the keto defects themselves. Indeed, the longer‐lived phosphorescence components (3.7 s and 680 ms for **MeLMMP** and **MeLPMP**, respectively) can be attributed to undisturbed π‐conjugated segments (pristine material in **MeLMMP**), where the formally forbidden ^3^π, π *→^1^π, π* transition leads to significantly longer emission lifetimes. In contrast, the shorter phosphorescence lifetime component is assigned to ladder‐type oligo(*meta*‐phenylene) segments adjacent to keto defects, where emission is partially quenched by fluorenone units (isolated 9‐fluorenone has a *T*_1_ energy of 2.19 eV [30], lower than the observed triplet energies of **MeLMMP** and **MeLPMP**).

Ultrafast time‐resolved transient absorption (fs‐TA) spectroscopy was performed on **MeLMMP** and **MeLPMP** in MCH. In both samples, fs‐TA measurements revealed a broad excited‐state absorption (ESA) band spanning from 400 to 800 nm (Figures S13 and S14). This ESA band exhibited a long‐lived decay component that extended beyond the temporal resolution of the fs‐TA equipment. To solve this, complementary nanosecond transient absorption (ns‐TA) measurements were performed, which confirmed the persistence of the same ESA band across the extended timescale.

**FIGURE 6:**
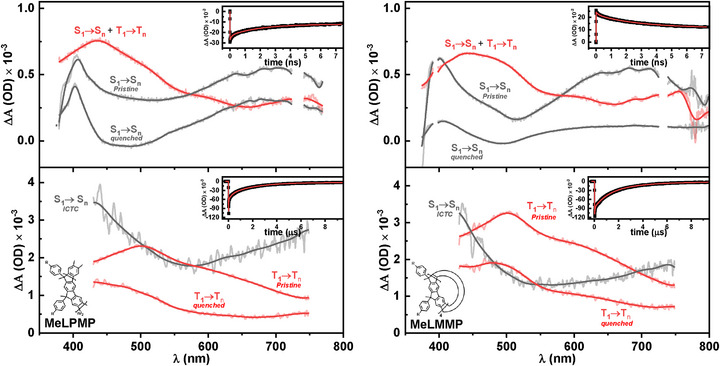
Decay‐associated spectra (DAS) of (left) **MeLPMP** and (right) **MeLMMP** in methylcyclohexane obtained from (top) femtosecond and (bottom) nanosecond transient absorption measurements. Each panel shows the spectral features with their corresponding process assignments. Insets display the decay traces of the principal kinetic components together with their respective fits. DAS containing triplet signatures are shown in red, and those with only singlet signatures in black, as a visual guide; (*R* = *n*‐decyl).

As shown in Figure 6, the decay‐associated spectra (DAS) for **MeLPMP** revealed two spectrally similar bands with lifetimes of approximately 120 ps and 2 ns. These are attributed to the pristine ladder‐type oligo(*meta*‐phenylene) and oligo(*meta*‐phenylene) chromophoric units electronically perturbed by proximity to keto defects, respectively. This assignment is supported by the agreement between these lifetimes and the fluorescence decay times (Table 2).

A third, broad spectral feature with a much longer lifetime is required to adjust the fs‐TA data fit. This long‐lived component was further investigated using nanosecond transient absorption (ns‐TA) spectroscopy. The DAS spectra (Figure 6) revealed three additional species in addition to the 120 ps and 2 ns components: two long‐lived species with lifetimes of approximately 2 µs and 270 ns, assigned to triplet transitions in pristine oligo(*meta*‐phenylene) units and in units influenced (quenched) by keto defects, respectively. This is again consistent with the bi‐exponential phosphorescence behavior observed. A third transient species exhibited a lifetime of approximately 13 ns. This last species is assigned to an ICTC between keto‐defective sites and adjacent oligo(*meta*‐phenylene) units, in agreement with the longest lifetime decay component found in the time‐resolved fluorescence experiments (Table 2). Similar results were obtained for **MeLMMP**, as shown in Figure 6, indicating that the *meta*‐phenylene ladder polymer and its macrocycle exhibit similar behavior. Therefore, the macromolecular system **MeLMMP** provides an ideal model for understanding the photophysics of the less‐explored *meta*‐phenylene polymer **MeLPMP**.

**SCHEME 2:**
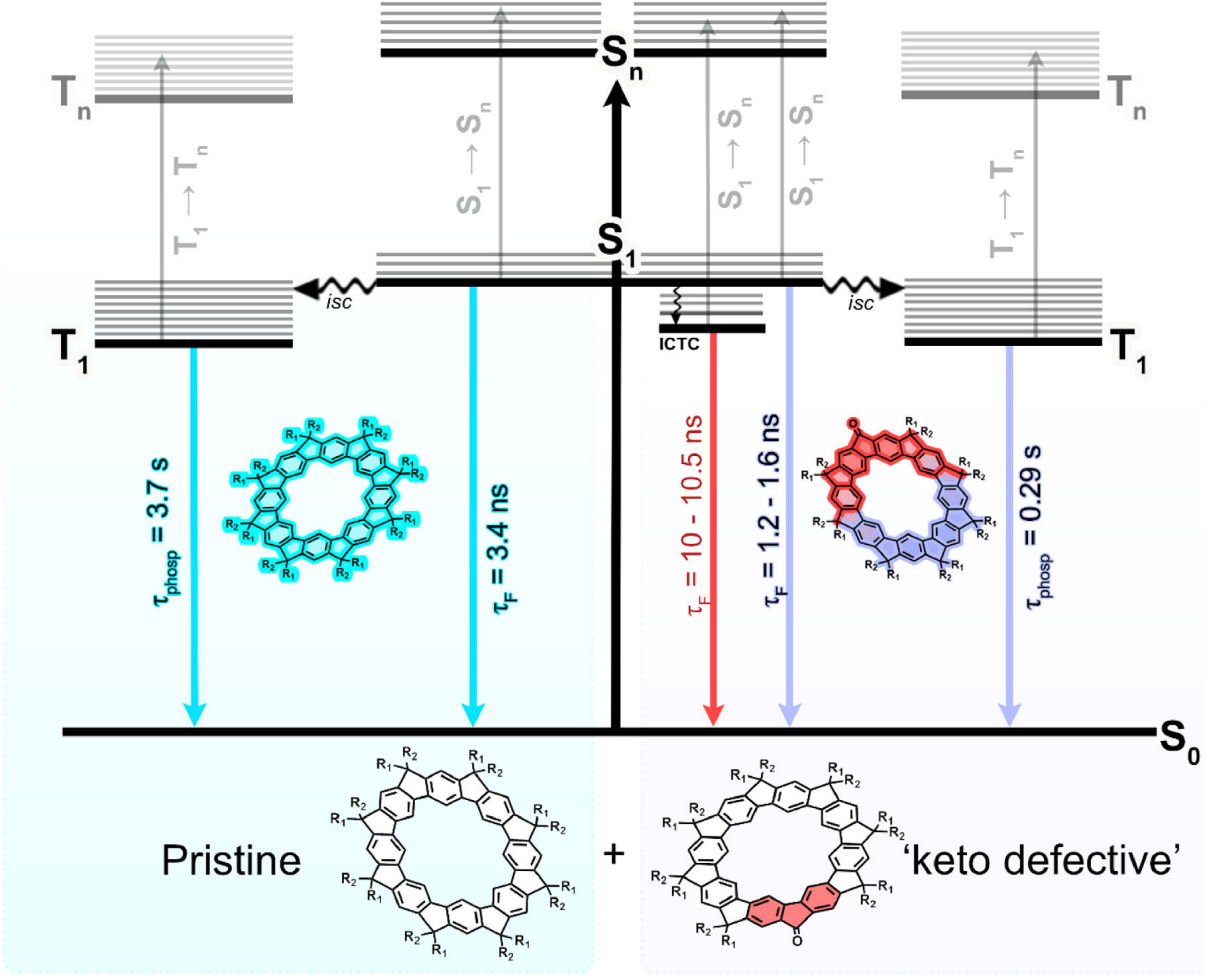
Jablonski‐type diagram illustrating the decay processes of the different chromophoric components in **MeLMMP**. The chromophores are color‐coded as follows: pristine **MeLMMP** (cyan), oligo(*meta*‐phenylene) units quenched by neighboring keto defects (purple), and intramolecular charge‐transfer complexes (ICTC) formed between keto defects and adjacent oligo(*meta*‐phenylene) units (red).

The proposed photophysical behavior for **MeLMMP** is depicted in Scheme 2, illustrating in a Jablonski‐type diagram the participation of multiple chromophoric species. A similar interpretation applies to **MeLPMP**.

In summary, the electronic behavior of the ladder‐type *meta*‐phenylene compounds **MeLMMP** and **MeLPMP** is influenced by fluorenone‐based defect sites, which modify the properties of neighboring oligo(*meta*‐phenylene) units. Time‐resolved fluorescence measurements revealed a dominant decay component of approximately 2 ns from pristine chromophoric segments, a shorter ∼1 ns component from units quenched by keto defects, and a longer‐lived component consistent with emission from ICTCs formed between defects and neighboring segments. These keto defects also account for the phosphorescence decay observed in both the macrocycle and polymer, as well as the mixture of species detected by transient absorption spectroscopy. Despite the complexity of the systems, the electronic and spectral features of each component could be systematically identified and distinguished, in agreement with previous reports on polyfluorene‐type copolymers containing fluorenone moieties [26].

## 3 Conclusion

A methyl‐substituted octa‐*meta*‐phenylene macrocycle (**MeLMMP**) and the corresponding ladder‐type poly(*meta*‐phenylene) (**MeLPMP**) were synthesized and thoroughly investigated. Both compounds exhibit comparable electronic behavior, indicating that the **MeLMMP** macrocycle, despite its lower number of phenylene units, closely mimics the properties of its ladder polymer counterpart.

Despite the photophysical complexity, the key fluorescence features were successfully assigned to distinct chromophoric environments within the macrocyclic and polymeric frameworks, highlighting the sensitivity of excited‐state behavior to subtle structural perturbations. Even in small amounts, keto defects influence the excited‐state dynamics of both **MeLMMP** and **MeLPMP**.

This study provides detailed photophysical and spectroscopic characterization of less‐explored *meta*‐phenylene ladder‐type systems. It highlights the potential of macrocyclic architectures as simplified models for understanding complex polymeric systems and lays the groundwork for the rational design of novel *meta*‐phenylene‐based macromolecular materials.

## Conflicts of Interest

The authors declare no conflicts of interest.

## Supporting information




**Supporting File 1**: The authors have cited additional references within the Supporting Information [31–43].
