# Supplementary material for: Chain Versus Ring: Characterization of a Meta‐Phenylene Ladder Polymer and Its Octameric Macrocycle
Source: Chemistry. 2026 Feb 9;32(16):e70744. doi: 10.1002/chem.70744 (PMC13109684; doi:10.1002/chem.70744)
Supplement: Supplementary file 1 — Supporting File 1: The authors have cited additional references within the Supporting Information [31–43]. [file CHEM-32-e70744-s001.pdf]

Supplementary information

Chain versus Ring: Characterization of a meta-Phenylene Ladder Polymer and Its Octameric Macrocycle

Paulo D. Nunes Barradas,[a] Hauke J. Jötten,[b] Ngoc B. B. Nguyen,[b] Ullrich Scherf,[b*] and J. Sérgio Seixas de Melo[a*]

[a]University of Coimbra, CQC-IMS, Department of Chemistry, Rua Larga, 3004-535 Coimbra, Portugal.

[b]Bergische Universität Wuppertal, Macromolecular Chemistry Group (buwmakro) and Wuppertal Center for Smart Materials and Systems (cm@s), Gauss-str. 20, D-42119, Wuppertal, Germany.

Table of contents:

MATERIALS AND METHODS **2**

Materials *2*
Experimental section *2*
Quantum electronic calculations *3*

SYNTHESIS **3**

MONOMER SYNTHESIS *3*
*General Ladderization Procedure Exemplified for the Polymer **MeLPMP*** *5*

SPECTROSCOPY DATA **8**

THEORETICAL CALCULATIONS **9**

TRANSIENT ABSORPTION **13**

REFERENCES **13**

Materials and Methods

Materials

All reactions were carried out under an argon atmosphere. All solvents used were either HPLC grade or as Acroseal bottles. Unless otherwise indicated, all reagents used were obtained from commercial suppliers and used without further purification.

For spectroscopic photoluminescence measurements, solvents, methylcyclohexane (MCH), and toluene, of spectroscopic grade were used.

Experimental section

Gel permeation chromatographic analysis (GPC) was carried out on an Agilent SECurity GPC System with two PPS SDV Linear S columns (5 µm particle size) and a PSS SDV pre-column (5 µm particle size). A G1362A RID and a G1315D DAD were used as detectors. All measurements were carried out at room temperature with THF as eluent and butylhydroxytoluene (BHT) as an internal standard using a polymer concentration of 1.0 g/L and a flow rate of 1.0 mL/min. The system was calibrated with polystyrene standards. A Shimadzu LC-System with an SPD-20AV UV detector was used for the preparative separation via recycling GPC. It contained a PSS SCV pre-column (5 µm particle size) as well as three PSS Linear S SCV columns (5 µm particle size). Separations were carried out with a concentration of 1.0 g/L at room temperature with THF as eluent (1.0 mL/min) and BHT as internal standard.

^{1}H and $^{13}C\{^{1}H\}$ NMR spectra were recorded either on a Bruker Avance 400 or an Avance III 600 spectrometer.

A field desorption mass spectrometer (FD-MS) from JOEL was used to determine the mass-to-charge ratio. The JMS-T200GC is equipped with an AccuTOF GCx.

Infrared spectra of solid samples were acquired using a Thermo Scientific Nicolet IS5 Fourier-Transform Infrared Spectrometer (FTIR) coupled to an ATR module model iD7. All measurements were conducted with 64 scans, employing a spectral resolution of 1 cm^{-1} with background subtraction.

UV-visible spectra were recorded using a double-beam spectrophotometer model Shimadzu UV-2700, covering the wavelength range from 200 - 800 nm and defining a slit width of 2 mm.

The emission and excitation spectra were recorded using a Jobin Yvon-Spex-Fluorolog 3-2.2 spectrofluorometer with correction of the instrumental response. The equipment was coupled to a thermal bath model Quantum and data acquisition was performed using a 90° geometry, with a time integration of 1 s and slits set at 1 nm. Any deviations from these conditions are noted in the main text. Fluorescence quantum yields (φ_F) in solution were determined by the comparative method using quinine sulphate as the reference material (φ_F = 0.546 in H_2SO_4 0.5 M).[31] The same equipment was used to record the phosphorescence emission and phosphorescence lifetimes of both samples. The excitation wavelength was set to 350 nm, and the emission wavelength was monitored at 500 nm

Fluorescence decay times (τ_F) were obtained using a custom-built nanosecond time-correlated single-photon counting (*ns*-TCSPC) setup with λ_{exc}=339 nm.[32-35] All measurements were conducted at 293 K in aerated conditions and the instrumental response was accounted by using a diluted scattering solution of colloidal silica (Ludox). The raw data was subjected to a global analysis at two different λ and modeled using a sum of discrete exponentials with the SAND program, employing the modulation functions method developed by Striker et al.[36] Distribution of the respective fluorescence lifetimes was also analyzed by the method of maximum entropy (MEM).[37-38]

Time-resolved ultrafast transient absorption (TA) measurements were carried out using a HELIOS broadband spectrometer (350 - 1600 nm, Ultrafast Systems), as described previously.[39] Samples were excited at 350 nm, and transient signals were recorded in the 400-800 nm spectral window. Measurements in solution were performed using a 2 mm quartz cuvette, with an optical density of approximately 0.3 at the excitation wavelength. To prevent photodegradation, samples were continuously translated using a motorized stage during data acquisition. Spectral chirp correction and global analysis of the TA data, including principal component analysis following singular value decomposition (SVD), were performed using the Surface Xplorer Pro software (Ultrafast Systems).

Nanosecond-millisecond transient absorption measurements were conducted using an EOS-Fire broadband spectrometer (350-1600 nm, Ultrafast Systems), which employs the same excitation source as the femtosecond setup. Excitation was also at 350 nm, and transient signals were monitored over the 400 - 800 nm range. Low excitation energy was used to avoid multiphoton excitation and triplet-triplet annihilation effects. Prior to measurement, all solutions were purged with nitrogen for at least 20 minutes to remove dissolved oxygen. Global analysis of the *ns*-TA data was carried out using the same software and approach as for the *fs*-TA data.

Quantum electronic calculations

Density Functional Theory (DFT) and time-dependent DFT (TDDFT) approaches were executed in the software GAMESS-US 2023R1.[40] Both DFT and TD-DFT calculations were conducted using a 6-311G(d,p) basis set and the B3LYP functional. In instances of $S_0 \rightarrow S_n$ transitions, a minor correction of 0.05 eV was introduced to rectify the variance between the zero-point and the initial vibronic level. Subsequently, optimized geometries were ascertained, followed by the execution of time-dependent DFT calculations (employing the identical functional and basis set as previous assessments) for forecasting the vertical electronic excitation energies. Furthermore, a frequency analysis was undertaken for each compound, revealing the absence of imaginary frequencies, signifying that the molecular structures correspond to, at the very least, a local energy minimum on the potential energy surface. Geometry optimization of a model simplified **MeLPMP** and all possible isomers of **MeLMMP** were obtained by performing PM6 semi-empirical calculations with the software MOPAC2016.[41-42]

Synthesis

Monomer Synthesis

The monomer **M2** was synthesized as described in the literature.[43]

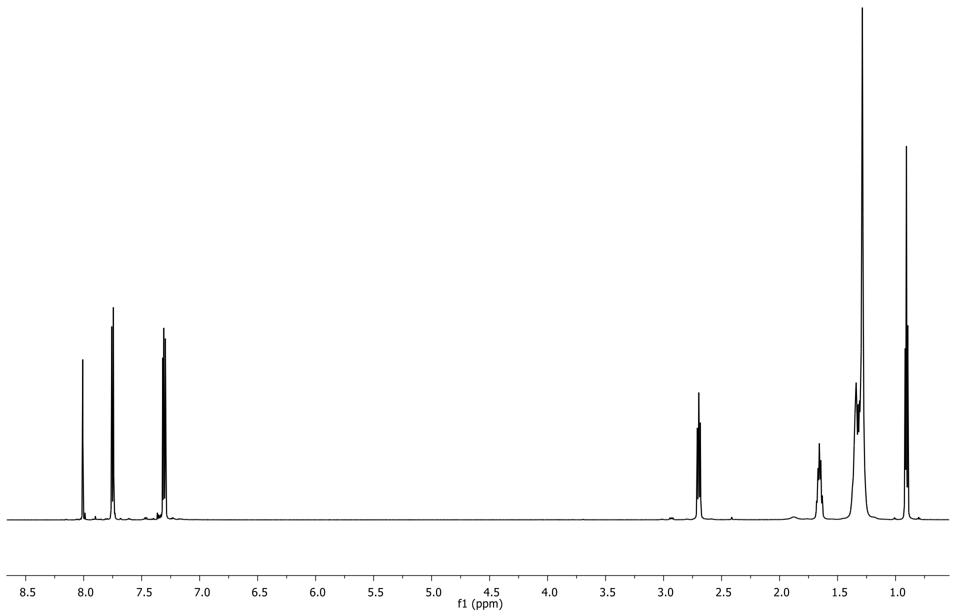

Figure S 1. ^{1}H NMR spectra of monomer **M2** (solvent: CDCl3)

Polymer and Macrocycle Synthesis (for comparison see Ref. [43]).

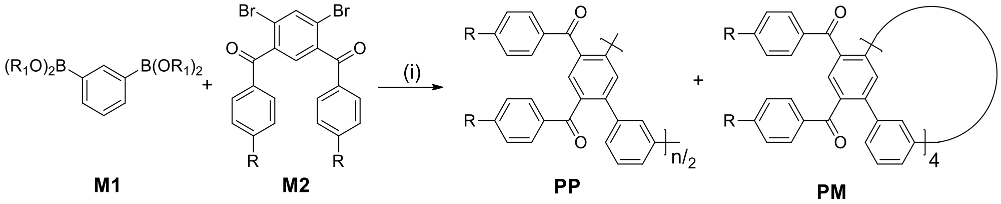

Scheme S 1. Synthesis of the **PP**/**PM** precursor mixture, (i) Pd(0)$(PPh_3)_4$, K_2CO_3, THF/water

Into six microwave vessels, diboronic ester **M1** (1 eq., 223 mg, 0.690 mmol), diketone **M2** (1 eq., 500 mg, 0.690 mmol), potassium carbonate (5 eq., 477 mg, 3.45 mmol) and tetrakis(triphenylphosphine) palladium(0) (0.05 eq., 39.9 mg, 0.035 mmol) were placed under argon atmosphere and dissolved in a mixture of 4.6 mL degassed water and 9.2 mL of tetrahydrofuran. The mixtures were then stirred at 75 °C for 72 h in the dark. After cooling to room temperature, the reaction products were combined, and chloroform and water added to the mixture. The organic phase was isolated, dried, and the solvents were then removed under reduced pressure. The product mixture was dissolved in a few drops of chloroform and the raw product isolated by precipitation into cold methanol. Afterward, the precipitate was purified via Soxhlet extraction with (1) methanol, (2) acetone, and (3) chloroform. The concentrated chloroform fraction was again precipitated into cold methanol. The GPC analysis showed two peaks, belonging to the precursor polymer **PP** (peak 2) and the precursor macrocycle **PM** (peak 1).

Table S 1. GPC analysis of the precursor mixture.

Peak	M_n [g mol^{-1}]	M_w [g mol^{-1}]	Đ
Entire sample	6,200	10,800	1.74
Only peak 1 (**PM**)	3,100	3,200	1.04
Only peak 2 (**PP**)	10,100	13,700	1.35

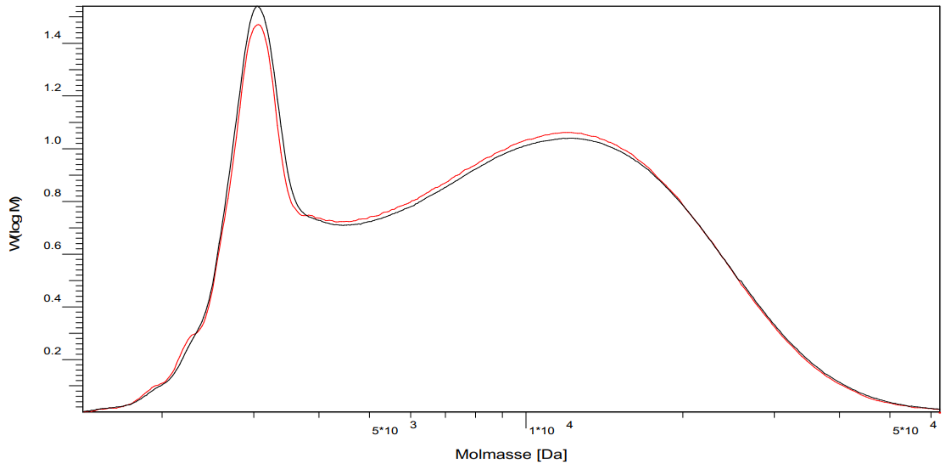

Figure S 2. GPC elugrams of the precursor mixture of **PP** and **PM** (chloroform fraction)

The two precursors **PM** and **PP** were then separated via recycling GPC, as shown in **Figure S 3**.

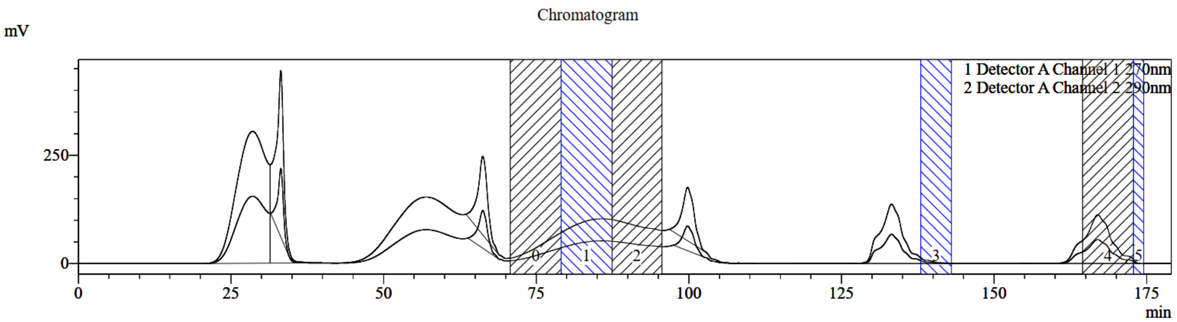

Figure S 3. Recycling GPC elugrams of the precursor mixture of **PP** (fraction 0, 1 and 2) and **PM** (fraction 4 and 5) (fraction 3 was not further considered).

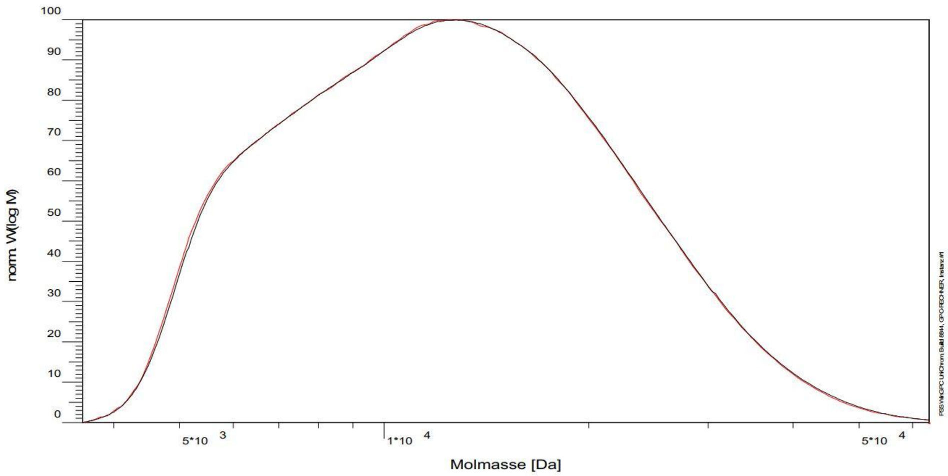

Figure S 4. GPC of the polymeric PP fraction.

Table S 2. Molecular weights of precursor polymer **PP** and macrocycle **PM**.

Compound	M_n [g mol^{-1}]	M_w [g mol^{-1}]	Đ	MW (FD mass spectrometry) [m/z]	Calculated mass [g/mol]	Yield[a] [mg]	Yield[a] [%]
PP	10,900	14,600	1.33	-	-	120	5
PM	-	-	-	2,561.7370	2,561.7116	300	11

[a] Final yield after recycling GPC purification.

*General Ladderization Procedure Exemplified for the Polymer **MeLPMP***

(1 eq., 300 mg, 0.8 mmol based on the molecular weight of the repeat unit) **PP** was dissolved in 55 mL of toluene under argon atmosphere. The microwave vessel was wrapped with aluminum foil, and methyllithium (18 eq., 1.6 mol L^{-1}, 5.20 mL, 8.3 mmol) was added dropwise at 0 °C. The reaction mixture was quenched with ethanol after 18 h stirring at room temperature. Afterwards, chloroform was added and the mixture washed with water and aqueous 2M HCl solution. The organic phase was concentrated and precipitated into cold methanol. The raw product was dissolved in 800 mL of dichloromethane, boron trifluoride etherate (30 eq., 2.90 mL, 23 mmol) was added and the mixture stirred for 18 h. The reaction was stopped by addition of ethanol. After washing with water and removing most of the solvent the product was isolated by precipitation into cold methanol.

The **MeLMMP** fraction was further purified by a second recycling GPC run.

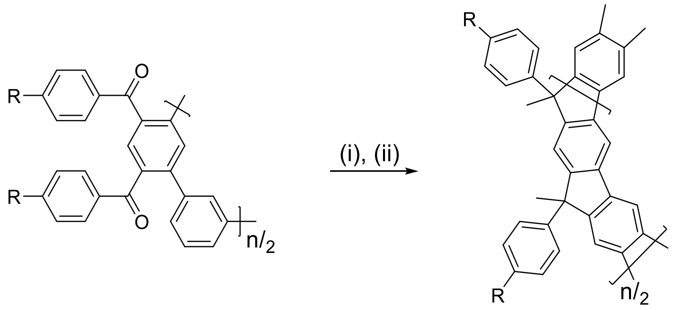

PP **MeLPMP**

Scheme S 2. Synthesis of polymeric **MeLPMP**; (i) MeLi/toluene; (ii) $BF_3 \cdot Et_2O$/dichloromethane

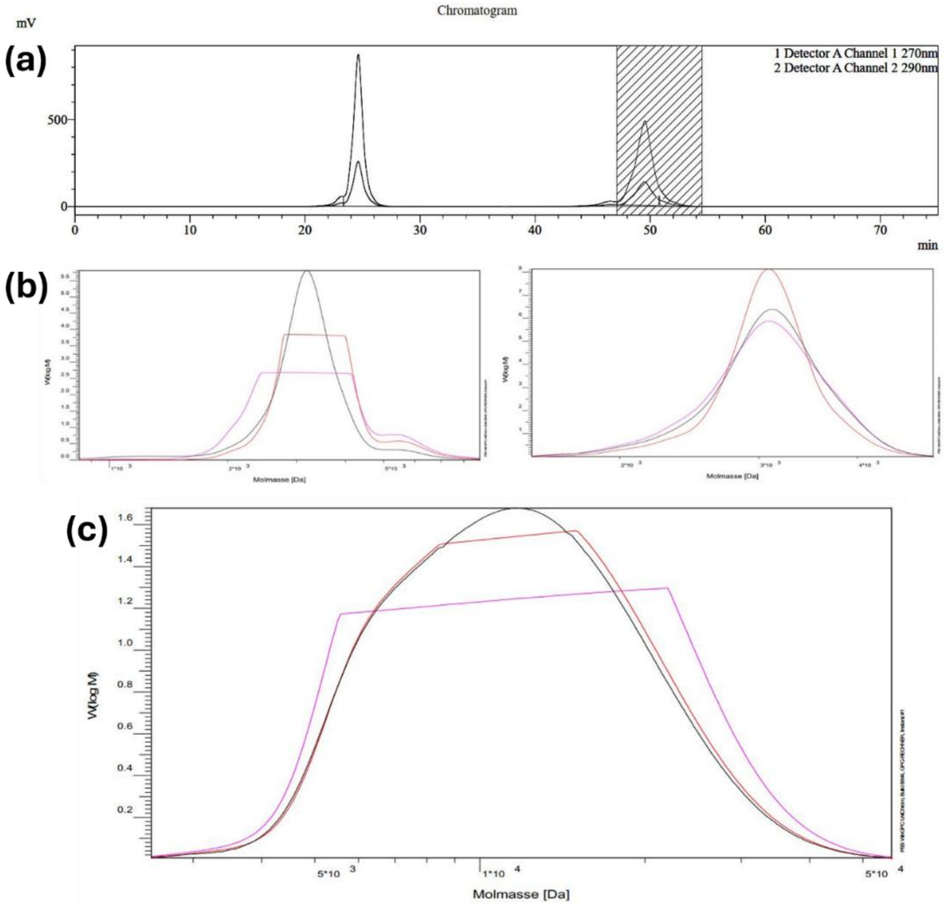

Figure S 5. (a) Recycling GPC elugrams of the macrocyclic MeLMMP. (b) GPC elugrams of the macrocyclic MeLMMP recorded before (left) and after (right) the second recycling GPC cycle. (c) GPC elugram of the polymeric MeLPMP.

Table S 3. GPC analyses and yields of **MeLPMP** and **MeLMMP** after post-polymerization ladderization

Compound	M_n [g mol^{-1}]	M_w [g mol^{-1}]	Đ	Yield[a] [mg]	Yield[a] [%]
MeLMMP	3,000	3,100	1.03	85	71
MeLPMP	10,200	13,000	1.28	266	89

[a] over two steps

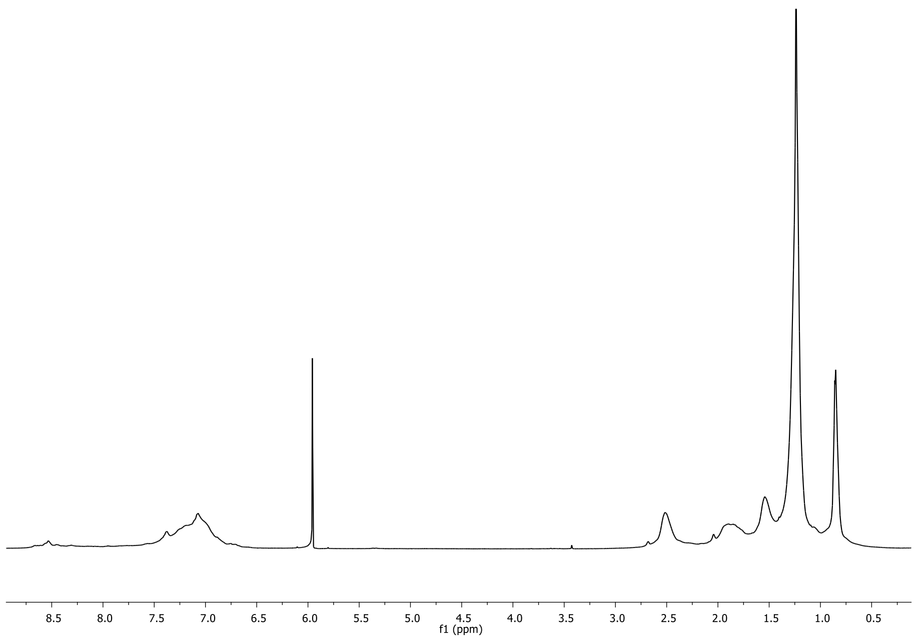

Figure S 6. ^{1}H NMR spectrum of **MeLMMP** (600 MHz, $C_2D_2Cl_4$, 300 K).

^{1}H NMR spectrum (600 MHz, $C_2D_2Cl_4$, 300 K) δ [ppm] = 8.81 – 6.32 (m, 12H), 2.82 – 2.28 (m, 4H), 2.09 – 1.66 (m, 4H), 1.63 – 1.38 (m, 5H), 1.39 – 0.95 (m, 27H), 0.95 – 0.40 (m, 8H).

^{13}C{^{1}H} NMR spectrum (151 MHz, $C_2D_2Cl_4$, 300 K) δ [ppm] = 153.2, 142.8, 141.1, 140.0, 128.6, 126.6, 122.1, 120.6, 112.2, 35.8, 32.2, 31.7, 30.0, 30.0, 29.9, 29.8, 29.7, 26.4, 23.0, 14.5.

FD MS [m/z]: 2,547.8472 (calculated mass: 2,547.8937)

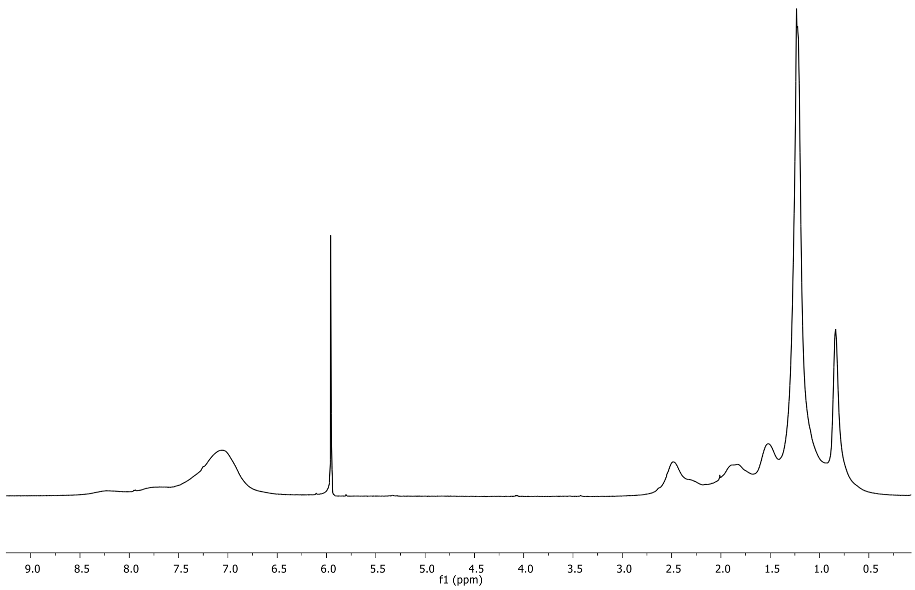

Figure S 7. ^{1}H NMR spectra of **MeLPMP** (600 MHz, $C_2D_2Cl_4$, 300 K).

^{1}H NMR spectrum (600 MHz, $C_2D_2Cl_4$, 300 K) δ [ppm] = 8.71 - 6.47 (m, 12H), 2.86 – 2.18 (m, 4H), 2.15 - 1.69 (m, 4H), 1.66 – 1.41 (m, 4H), 1.41 – 0.95 (m, 22H), 0.92 – 0.43 (m, 6H).

$^{13}C\{^1H\}$ NMR spectrum (151 MHz, $C_2D_2Cl_4$, 300 K) δ [ppm] = 154.5, 142.9, 141.0, 139.5, 128.5, 126.6, 123.9, 123.1, 120.6, 54.4, 51.8, 35.8, 32.2, 31.6, 30.0, 29.9, 29.8, 29.6, 23.0, 14.5.

Table S 4. Molecular weights of the polymer **MeLPMP** and the macrocycle **MeLMMP**.

Compound	M_n [g mol^{-1}]	M_w [g mol^{-1}]	Đ	Molecular weight (FD mass spectrometry) [m/z]	Calculated mass [g/mol]
Precursor mixture	6,200	10,800	1.74	-	-
PP	10,900	14,600	1.33	-	-
PM	-	-	-	2,561.7370	2,561.7116
MeLPMP	10,200	13,000	1.28	-	-
MeLMMP	3,000	3,100	1.03	2,547.8472	2,547.8937

Spectroscopy data

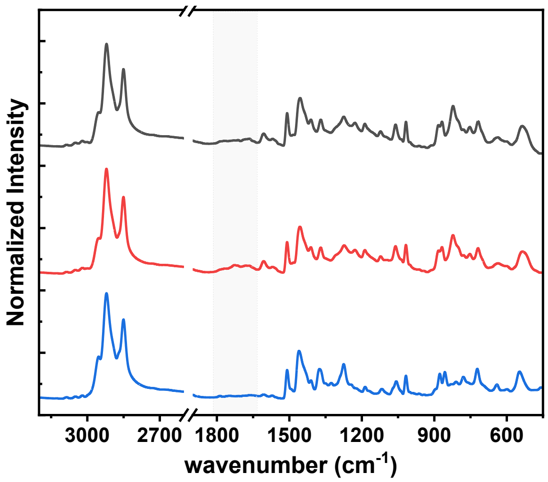

Figure S 8. FTIR spectra of (black) **MeLPMP** and (red) **MeLMMP**. As a reference, the spectrum of ladder poly(*para*-phenylene) (**MeLPPP**) is shown in blue.

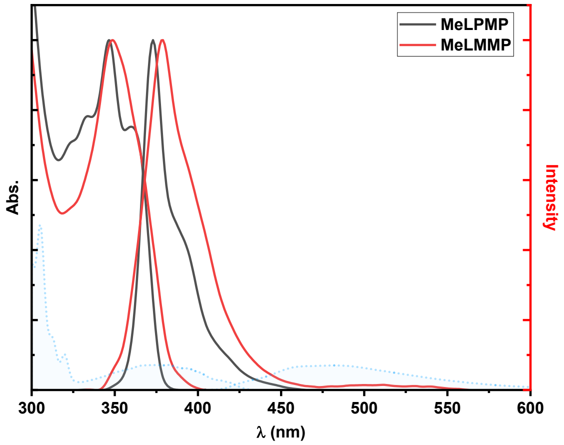

Figure S 9. Absorption and fluorescence emission spectra at 293 K, λ_{exc}=310nm, of MeLPMP (black lines) and MeLMMP (red lines) in toluene. Spectra of 9-fluorenone (dashed blue) recorded under the same conditions are included as a reference for identifying keto-defect signatures.

Statistical approach

Data analysis was performed using Python scripts available at the following repository: https://github.com/paulodnb/MeLMMP-isomers. Images of all unique structural isomers are provided in the same directory, with the number of times each structure is repeated to complete all possible permutations indicated as

a superscript. For a system with 38 elements, assuming equal event probabilities, the total number of combinations is 6561. However, for the MeLMMP system, the C_{8v} symmetry of the macrocycle relative to its center reduces this to 834 unique structures, of which only 36 are defect-free.

When these 834 structures are categorized by their number of defects, a frequentist histogram of the probability of keto-defect occurrence can be constructed (Figure S10). This indicates that in a completely random process where keto-defects have an equal probability of occurring, the stereoisomer population for MeLMMP should be dominated by structures containing 2 to 3 defects. Under actual experimental conditions, however, the formation of keto-defects is an undesirable side reaction; consequently, the major population of stereoisomers is expected

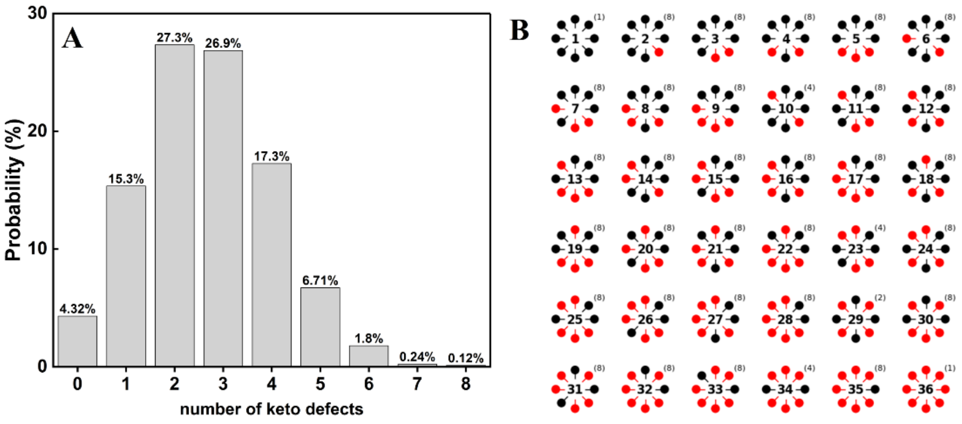

Figure S 10. (A) Histogram of frequentist probability of possible keto defects in **MeLMMP** structure. (B) Schematical representation of all 36 unique isomers for an ideal macrocycle without keto defects from a top view; black points represent methyl groups and red points represent phenyl groups. In the case of ideal ladder isomers, those with a 1:1 methyl: phenyl ratio have the highest probability.

Theoretical calculations

A semi-empirical optimization at the PM6 level of theory was performed for all 36 unique isomers of **MeLMMP**, excluding the C10-alkyl side chains for practical purposes. As shown in Table S5, the most stable structures are those with the least steric hindrance, specifically structure 29, where all phenyl groups are in anti-position relative to each other.

DFT optimizations and TDDFT predictions for the excited states were performed on the most stable structure predicted by the PM6 method (structure 29). The B3LYP/6-311G(d,p) level of theory provided the closest match to the lowest energy band observed in the experimental data. However, semi-empirical predictions of the excited state using the PM6 method for the same structure also closely resembled the experimental data, as seen in the **Figure S 12**.

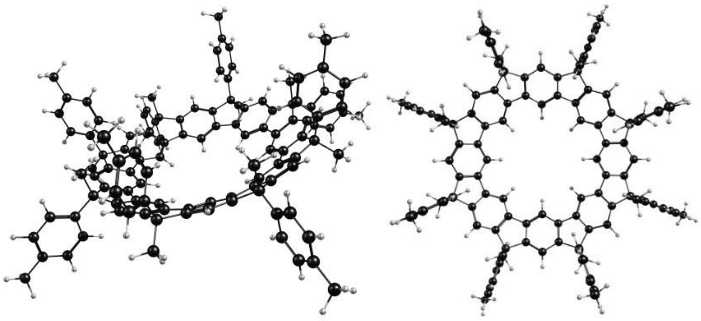

Figure S 11. Model Structures proposed for **MeLMMP** used for DFT and TDDFT calculations.

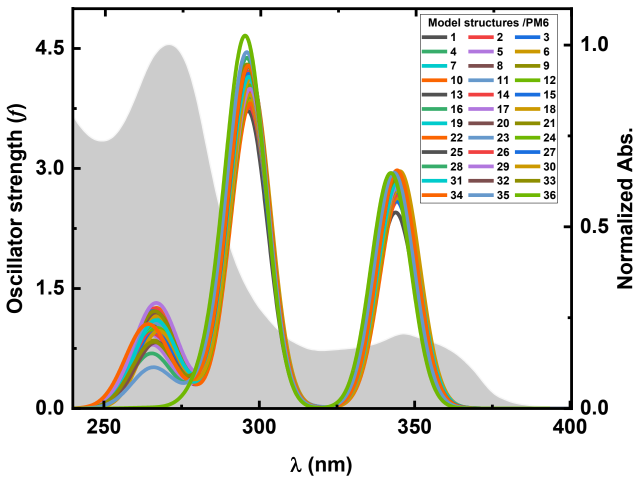

Figure S 12. (A) Predicted absorption spectra for all 36 **MeLMMP** isomer models listed in **Table** S1 using PM6 INDO/ CIS level. A Gaussian fit was used to simulate the absorption spectrum.

Table S 5. Energy and optimized structures of all possible unique isomers of **MeLMMP** at PM6 semi-empirical level of theory.

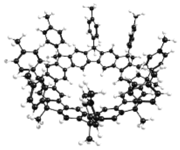<br>(1)<br>13.989 eV	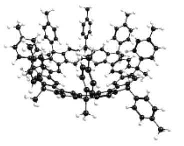<br>(2)<br>13.988 eV	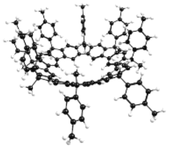<br>(3)<br>13.988 eV	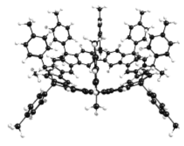<br>(4)<br>13.974 eV	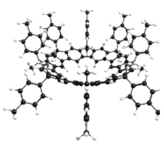<br>(5)<br>13.994 eV	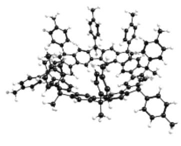<br>(6)<br>13.974 eV
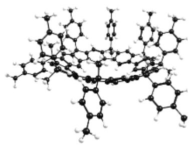<br>(7)<br>13.977 eV	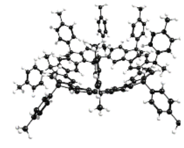<br>(8)<br>13.980 eV	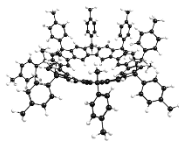<br>(9)<br>13.993 eV	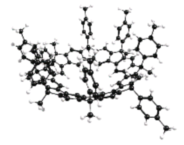<br>(10)<br>13.981 eV	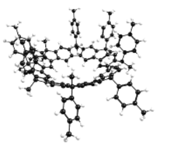<br>(11)<br>13.977 eV	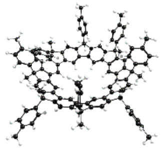<br>(12)<br>13.967 eV
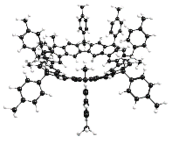<br>(13)<br>13.986 eV	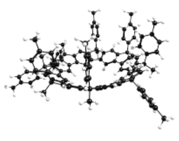<br>(14)<br>13.977 eV	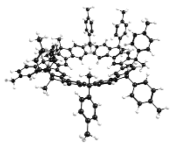<br>(15)<br>13.980 eV	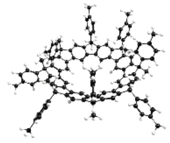<br>(16)<br>13.986 eV	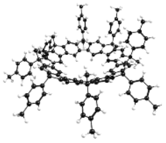<br>(17)<br>13.998 eV	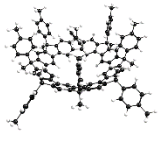<br>(18)<br>13.962 eV
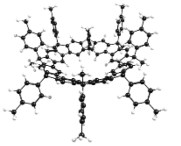<br>(19)<br>13.986 eV	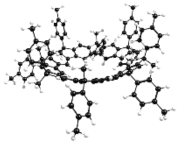<br>(20)<br>13.966 eV	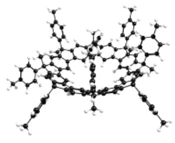<br>(21)<br>13.966 eV	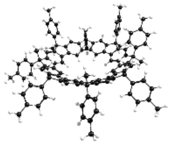<br>(22)<br>13.984 eV	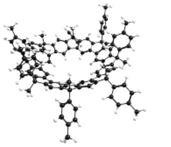<br>(23)<br>13.979 eV	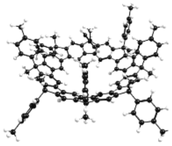<br>(24)<br>13.964 eV
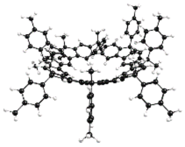<br>(25)<br>13.986 eV	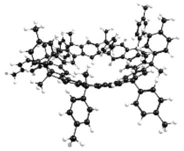<br>(26)<br>13.986 eV	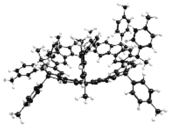<br>(27)<br>13.984 eV	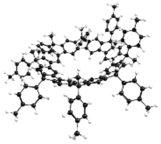<br>(28)<br>14.007 eV	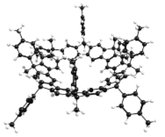<br>(29)<br>13.952 eV	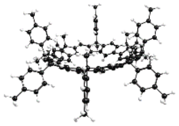<br>(30)<br>13.978 eV
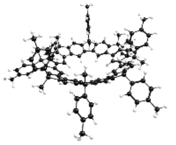<br>(31)<br>13.972 eV	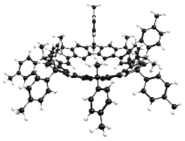<br>(32)<br>13.990 eV	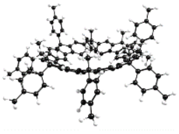<br>(33)<br>13.984 eV	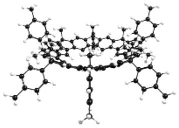<br>(34)<br>13.992 eV	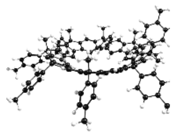<br>(35)<br>14.002 eV	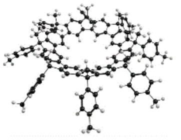<br>(36)<br>14.016 eV

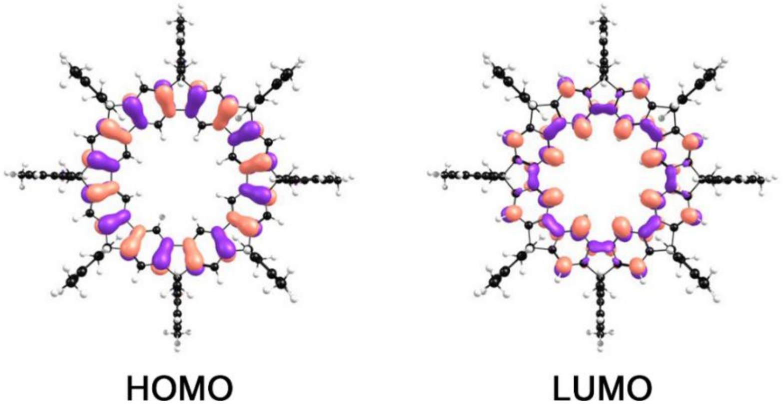

Figure S 13. Graphical representation of the HOMO and LUMO orbitals of the model **MeLMMP** structure, calculated at the B3LYP/6-311G(d,p) level of theory.

Table S 6. Predicted (by TDDFT) wavelength absorption maxima and oscillator strengths (*f*) for the first 20 excited states of the pristine **MeLMMP** model and those containing keto defects. The **Table** includes graphical representations of the orbitals involved in the most significant transitions for each state.

Compound Models	ΔE_{L-H}	state	Energy /eV	f	λ_{abs}*/nm	Electronic transition**
MeLMMP		1	3.48	5×10^{-6}	362	HOMO → LUMO
		2	3.74	0.6763	336	HOMO -1 → LUMO
		3	3.74	0.6722	336	HOMO → LUMO +2
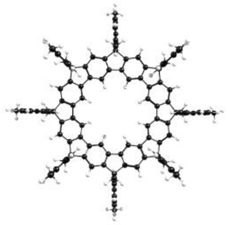		4	3.77	0.0001	333	HOMO → LUMO +1
		5	3.92	0.0008	320	HOMO → LUMO +4
		6	3.92	0.0009	320	HOMO → LUMO +5
		7	3.95	0.0057	318	HOMO -1 → LUMO
(top view)		8	3.95	0.0046	318	HOMO -2 → LUMO
		9	3.97	1.7×10^{-5}	316	HOMO -1 → LUMO +2
	4.06	10	3.97	$8. \times 10^{-6}$	316	HOMO -1 → LUMO +3
		11	3.98	1.4×10^{-5}	316	HOMO -1 → LUMO +1
		12	3.98	2×10^{-6}	316	HOMO -2 → LUMO +1
		13	4.05	0.0006	310	HOMO -1 → LUMO +4
		14	4.05	0.0004	310	HOMO -2 → LUMO +2
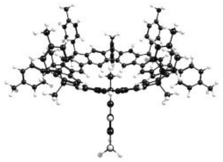		15	4.13	0.0060	304	HOMO -1 → LUMO +2
		16	4.15	0.0002	303	HOMO → LUMO +6
(side view)		17	4.16	$4. \times 10^{-5}$	302	HOMO → LUMO +7
		18	4.19	2.1×10^{-5}	300	HOMO -2 → LUMO +4
		19	4.21	1.1×10^{-5}	298	HOMO → LUMO +6
		20	4.23	$5. \times 10^{-6}$	297	HOMO → LUMO +7

* Predicted maxima absorption value from TDDFT.**Electronic transition with higher contribution to the respective state.

Transient absorption

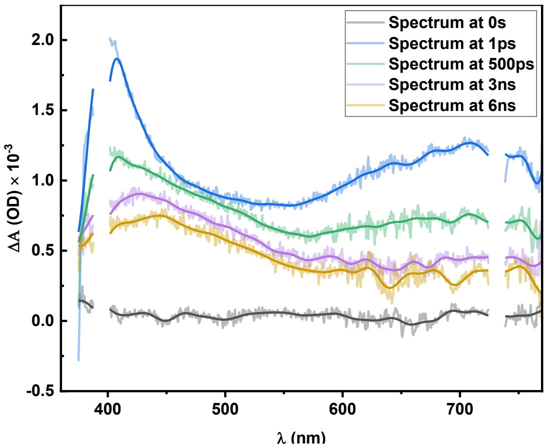

Figure S 14. Femtosecond transient absorption spectra of **MeLPMP** in methylcyclohexane (MCH) at selected time delays following excitation at 350 nm. The spectra were recorded in the 400-800 nm range using a broadband pump-probe setup.

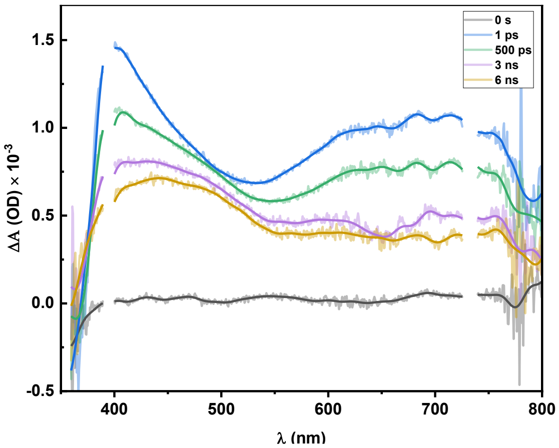

Figure S 15. Femtosecond transient absorption spectra of **MeLMMP** in methylcyclohexane (MCH) at selected time delays following excitation at 350 nm. The spectra were recorded in the 400-800 nm range using a broadband pump-probe setup.

References

[31] D. F. Eaton,"Reference materials for fluorescence measurement", *Pure and Applied Chemistry* **1988**, *60*, 1107-1114.

[32] J. Seixas de Melo,"The influence of oxygen on the lifetime of luminescent probes. A simple device for degassing solutions for fluorescence measurements", *Chem. Educ* **2005**, *10*, 29-35.

[33] J. Pina, J. Seixas de Melo, H. D. Burrows, A. L. Maçanita, F. Galbrecht, T. Bunnagel, U. Scherf,"Alternating Binaphthyl-Thiophene Copolymers: Synthesis, Spectroscopy, and Photophysics and Their Relevance to the Question of Energy Migration versus Conformational Relaxation", *Macromolecules* **2009**, *42*, 1710-1719.

[34] J. S. Seixas de Melo, J. Pina, F. B. Dias, A. L. Maçanita,"Experimental techniques for excited state characterisation", *Applied photochemistry* **2013**, 533-585.

[35] A. C. B. Rodrigues, A. Eckert, J. Pina, U. Scherf, J. S. Seixas de Melo,"Polymeric near infrared emitters with bay-annulated indigo moieties", *Materials Advances* **2021**, *2*, 3736-3743.

[36] G. Striker, V. Subramaniam, C. A. M. Seidel, A. Volkmer,"Photochromicity and Fluorescence Lifetimes of Green Fluorescent Protein", *The Journal of Physical Chemistry B* **1999**, *103*, 8612-8617.

[37] M. N. Berberan-Santos, B. Valeur,"Luminescence decays with underlying distributions: General properties and analysis with mathematical functions", *Journal of Luminescence* **2007**, *126*, 263-272.

[38] J. M. Shaver, L. B. McGown,"Maximum entropy method for frequency domain fluorescence lifetime analysis. 1. Effects of frequency range and random noise", *Anal Chem* **1996**, *68*, 9-17.

[39] J. Pina, M. J. Queiroz, J. Seixas de Melo,"Effect of substitution on the ultrafast deactivation of the excited state of benzo[b]thiophene-arylamines", *Photochem Photobiol Sci* **2016**, *15*, 1029-1038.

[40] G. M. J. Barca, C. Bertoni, L. Carrington, D. Datta, N. De Silva, J. E. Deustua, D. G. Fedorov, J. R. Gour, A. O. Gunina, E. Guidez, T. Harville, S. Irle, J. Ivanic, K. Kowalski, S. S. Leang, H. Li, W. Li, J. J. Lutz, I. Magoulas, J. Mato, V. Mironov, H. Nakata, B. Q. Pham, P. Piecuch, D. Poole, S. R. Pruitt, A. P. Rendell, L. B. Roskop, K. Ruedenberg, T. Sattasathuchana, M. W. Schmidt, J. Shen, L. Slipchenko, M. Sosonkina, V. Sundriyal, A. Tiwari, J. L. Galvez Vallejo, B. Westheimer, M. Wloch, P. Xu, F. Zahariev, M. S. Gordon,"Recent developments in the general atomic and molecular electronic structure system", *J Chem Phys* **2020**, *152*, 154102.

[41] J. J. P. Stewart, Version, **2016**.

[42] J. J. P. Stewart,"Optimization of parameters for semiempirical methods V: Modification of NDDO approximations and application to 70 elements", *Journal of Molecular Modeling* **2007**, *13*, 1173-1213.

[43] U. Scherf, K. Muellen,"Poly(arylenes) and poly(arylenevinylenes). 11. A modified two-step route to soluble phenylene-type ladder polymers", *Macromolecules* **1992**, *25*, 3546-3548.
